# Antipsychotic Polypharmacy and High-Dose Antipsychotic Regimens in the Residential Italian Forensic Psychiatric Population (REMS)

**DOI:** 10.3389/fpsyg.2022.722985

**Published:** 2022-02-10

**Authors:** Gabriele Mandarelli, Felice Carabellese, Guido Di Sciascio, Roberto Catanesi

**Affiliations:** ^1^Interdisciplinary Department of Medicine, Section of Criminology and Forensic Psychiatry, University of Bari “Aldo Moro”, Bari, Italy; ^2^Department of Mental Health, Bari, Italy

**Keywords:** REMS, antipsychotic polypharmacy (APP), high-dose antipsychotics, schizophrenia spectrum disorders, personality disorders, forensic psychiatric treatment

## Abstract

Few data exist regarding treatment with antipsychotics in forensic psychiatric patient populations with high social dangerousness. We performed a secondary analysis of 681 patients treated with at least one antipsychotic, extracted from a 1-year observational retrospective study, conducted on 730 patients treated in the Italian Residencies for Execution of Security Measures (REMS) (96.4% of the REMS population). We aimed at investigating antipsychotic polypharmacy (prescription of two or more concomitant antipsychotics) and high dose/very high-dose antipsychotics, as well as the possible factors associated with such therapeutic regimens. High dose/very high-dose antipsychotics were defined as a prescribed daily dose to WHO-defined daily dose ratio greater than 1.5 or 3.0, respectively. Binary logistic regression analysis was used in three models to test possible predictors of antipsychotic polypharmacy, high-dose antipsychotics, and very high-dose antipsychotic prescription. Antipsychotic polypharmacy resulted in *n* = 308 (45.2%) of the patients, *n* = 346 (50.8%) received high-dose antipsychotics, and *n* = 96 (14.1%) very high-dose antipsychotics. The multivariate analysis disclosed an association between antipsychotic polypharmacy and male gender (odds ratio (OR): 2.75 and 95% CI: 1.34–5.65), long-acting injectable (LAI) antipsychotic prescription (OR: 2.62 and 95% CI: 1.84–3.74), and aggressive behavior in REMS (OR: 1.63 and 95% CI: 1.13–2.36). High-dose antipsychotics were also associated with male gender (OR: 2.01 and 95% CI: 1.02–3.95), LAI antipsychotic prescription (OR: 2.78 and 95% CI: 1.95–3.97), and aggressive behavior in REMS (OR: 1.63 and 95% CI: 1.12–2.36). The use of antipsychotic polypharmacy and high-dose antipsychotics is frequent in the REMS population. These results might depend on regulatory and organizational aspects of the REMS system, including variability in structures, lack of a common model of care, and lack of stratified therapeutic security.

## Introduction

In recent years, Italy has undergone a radical change in the healthcare system aimed at treating psychiatric patients who have committed crimes and who are also considered socially dangerous by a judicial measure (Barbui and Saraceno, 2015; Carabellese and Felthous, 2016). The reform provided for the closure of the former six forensic psychiatric hospitals and the opening of small regional-based residential structures called Residencies for Execution of Security Measures (REMS; Catanesi et al., 2019).

The 31 existing REMS constitute a pure healthcare system, devoid of police personnel, and aimed at combining the needs of care of patients, with those of social security (Barbui and Saraceno, 2015; Carabellese and Felthous, 2016). Unlike other legal systems, forensic psychiatric patients admitted to the REMS have no treatment obligations despite being compulsorily admitted. Moreover, the Italian Law does not provide social dangerousness as a possible criterion for involuntary psychiatric treatment in civil nor in forensic psychiatric patients (Carabellese and Mandarelli, 2017; Ferracuti et al., 2020). This implies that patients are obliged to remain physically in the REMS but not to take treatments for which they must provide their informed consent.

A recent 1-year study on the Italian forensic psychiatric population revealed that among the 730 patients who have been treated in the REMS, 60% suffered from a schizophrenia spectrum disorder, 32% by a personality disorder, and there was relevant comorbidity with substance-related disorders (Catanesi et al., 2019). In this context, the psychopharmacological choices made by psychiatrists deserve interest because they need to combine different and specific needs of dangerous offenders affected by severe mental disorders, with safety ones.

Antipsychotic polypharmacy and high-dose antipsychotic prescription regimens have been reported in acute psychiatric settings (Lelliott et al., 2002; Connolly and Taylor, 2014; John et al., 2014; Campos Mendes et al., 2016; McMillan et al., 2017) and community settings (Callaly and Trauer, 2000; Gisev et al., 2014a). The use of depot antipsychotics proved more frequent in those patients with a community treatment order (Callaly and Trauer, 2000; Gisev et al., 2014a). Antipsychotic polypharmacy and high-dose antipsychotics are therapeutic strategies with greater impact, and they are often unaligned to the prescribing clinical guidelines, burdened by greater side effects, possible drug interactions, and the risk for complete non-adherence (Cullen et al., 2013; Papola et al., 2019; Smith et al., 2020; Aburamadan et al., 2021; Lochmann van Bennekom et al., 2021).

However, a systematic review and meta-analysis showed comparable mortality risk between patients with serious mental illness treated with antipsychotic polypharmacy vs. monotherapy (Buhagiar et al., 2020). A lack of association between high dose or combination antipsychotics and side effects has been reported on a mixed-diagnosis sample of 208 inpatients (Hynes et al., 2020). There is also initial evidence that antipsychotic combination therapy might prevent rehospitalization in a subset of patients with schizophrenia (Faden et al., 2021).

In a study on 211 patients with psychoses in a community psychiatric setting, 17.5% received two antipsychotics and 13.7% were on a high-dose antipsychotic regime (Tungaraza et al., 2010). Approximately one-third of compulsorily treated community patients suffering from psychoses received antipsychotic polypharmacy and 27% high-dose antipsychotics in a retrospective Australian study (Gisev et al., 2014b). Involuntarily treated psychiatric patients under the Mental Health Act were 8.8 more likely than their voluntary counterparts to receive antipsychotic polypharmacy and 1.65 to receive high doses of antipsychotics (Wheeler et al., 2020). Forensic psychiatric settings have been associated with greater use of antipsychotic polypharmacy, high dose, or both than acute or rehabilitation settings (Lelliott et al., 2002).

Based on the limited existing data, we aimed this study at describing the pharmacological choices of the REMS psychiatrists and at identifying the factors associated with antipsychotic polypharmacy and high-dose antipsychotics in a large cohort of patients who were admitted and treated in the Italian REMS.

## Materials and Methods

We did a secondary analysis of the 681 patients who received at least an antipsychotic prescription, among those extracted from an observational retrospective study of 730 patients treated in the Italian REMS in the 1-year study period (Catanesi et al., 2019). The original study described the clinical, criminological, and treatment characteristics of the REMS patient population between June 2017 and June 2018. Data were retrieved by the health managers of the 28 participating REMS through an *ad hoc* form (Catanesi et al., 2019). The 49 patients who were excluded because not under antipsychotic treatment were less frequently affected by schizophrenia spectrum disorders (*p* < 0.001) while presented a higher percentage of personality disorder diagnosis (*p* < 0.001) than their antipsychotic treated counterparts. The presence and degree of aggressive behavior of patients were measured with the Italian version of the Modified Overt Aggression Scale (MOAS; Margari et al., 2005).

Antipsychotic polypharmacy was defined as the prescription of two or more concomitant antipsychotics in the standard therapy of a patient. We used the WHO Collaborative Center for Drug Statistics Methodology Defined Daily Dose (DDD; WHO Collaborating Centre for Drug Statistics Methodology, 2021) method to allow comparison among different antipsychotics (Leucht et al., 2016) and calculated the prescribed daily dose (PDD) to DDD ratio (Roh et al., 2014). To have a comprehensive measure of the antipsychotic dosage for each patient, we summed the PDD to DDD ratio for each antipsychotic taken. In this study, the PDD/DDD ratio will be considered as the sum of the total ratios of the prescribed antipsychotics, both oral and long-acting injectable (LAI). To calculate the PDD of the LAI antipsychotics, we divided the prescribed dose by the dosing interval. Based on previous research, we considered high antipsychotic dose as a PDD to DDD ratio of greater than 1.5 (Roh et al., 2014). We have also introduced a higher threshold, intended as a very high antipsychotic dose that was defined as a PDD/DDD greater than 3.0.

### Statistical Analysis

Data were analyzed using Statistical Software for Social Sciences (SPSS) version 20.0. The chi-square test or Fisher’s exact test was used for comparisons between categorical variables. Mann–Whitney *U* test was used to test differences between continuous non-parametric variables. Binary logistic regression analysis was used in three models to test possible predictors of antipsychotic polypharmacy, high-dose antipsychotics (PDD/DDD > 1.5), and very high-dose antipsychotic prescription (PDD/DDD > 3). We used the enter method to test the following independent variables: male gender, LAI antipsychotic prescription, diagnosis of schizophrenia spectrum disorder and other psychoses, diagnosis of personality disorder, length of stay in REMS, and aggressive behavior in REMS in the previous month (MOAS > 0) (Catanesi et al., 2019). For each model Cox & Snell R2, odds-ratio (OR) estimates and their corresponding 95% CI are reported.

## Results

The study of *n* = 681 patient sample (*M*_age_ = 41.4 years and SD 11.6) comprised 89.4% of male and 10.6% female patients, and the main sociodemographic and clinical characteristics are shown in Table 1. Notably, 65% of the patients had a principal psychiatric diagnosis of schizophrenia spectrum and other psychotic disorders (35.4% schizophrenia, 13.5% unspecified schizophrenia spectrum disorder and other psychotic disorder, 8.5% delusional disorder, and 7.6% schizoaffective disorder), 21% of personality disorder (of which 75.6% main diagnosis and 24.4% comorbid diagnosis), and 22.7% of the patients presented a comorbid diagnosis of substance-related disorder.

**TABLE 1:** Sociodemographic and clinical characteristics of *n* = 681 patients.

Age, years, mean (SD); range	41.4 (11.6); 19–80
Males, %	89.4%
Caucasian, %	85.5%
Principal psychiatric diagnosis^1^, %	
Schizophrenia	34.5%
Personality disorders	21.4%
Unspecified schizophrenia spectrum and other psychotic disorder	12.8%
Bipolar I disorder	9.3%
Delusional disorder	8.2%
Schizoaffective disorder	7.6%
Neurodevelopmental disorders	2.9%
Other	3.3%
Disease duration, years, mean (SD); range	14.5 (9.6); 0.5–42.0
Length of stay in REMS, months, mean (SD); range	14.5 (10.0); 1.0–39.0
Already in care at public mental health services,%	82.4%
Previous psychiatric admission,%	73.1%
Previous involuntary psychiatric admission,%	59.3%
History of substance use,%	57.1%
Aggressive behavior in REMS, previous month (MOAS > 0),%	35.8%

*^1^Given the high rate of comorbid diagnosis, these data refer to the previous axis I of the DSM-IV-TR.*

The analysis of crimes underlying the admission to the REMS revealed 26.5% of homicide/attempted homicide, 24.4% personal injury/threats/harassment, 17.7% domestic violence, 12.3% property crime, 7.5% violence against a public official, 6.5% stalking, 4.0% sexual offenses, and 1.1% misdemeanors. Patients had a long mean disease duration (14.5 ± 9.6 years) and a history of previous psychiatric hospitalization including involuntary admission (59.3%).

The main drug treatment characteristics as well as the associations between antipsychotics and other drugs are described in the original study (Catanesi et al., 2019). The most frequent combination of antipsychotics we found in this study was haloperidol decanoate and oral olanzapine, haloperidol decanoate and quetiapine, paliperidone palmitate and clozapine, oral haloperidol and clozapine, and oral haloperidol and oral olanzapine.

Among the 681 REMS patients, *n* = 308 (45.2%) were prescribed antipsychotic polypharmacy, *n* = 346 (50.8%) high-dose antipsychotics, and *n* = 96 (14.1%) very high-dose antipsychotics.

Among those patients treated with antipsychotic polypharmacy, 79.9% also received high-dose antipsychotics vs. 26.8% of those in monotherapy (*p* < 0.001), and 28.2% received very high-dose antipsychotics vs. 2.4% of those in monotherapy (*p* < 0.001). Chi-square test showed that male patients more frequently received antipsychotic polypharmacy (men 47.1%, women 29.2%; *p* < 0.01) and very high-dose antipsychotics (men 15.1%, women 5.6%; *p* < 0.05) than female patients, while we found no significant gender differences in high-dose antipsychotics (men 51.9%, women 41.7%; *p* = 0.10).

The mean PDD/DDD ratio of the 681 patients was 1.94 (SD: 1.56 and 95% CI: 1.75–1.99), median 1.62, and ranged from a minimum of 0.02 to a maximum of 21.39. Mann–Whitney *U* test disclosed no significant differences in the mean PDD/DDD ratio between male and female patients (men 1.96 ± 1.61, women 1.73 ± 1.38; *p* = 0.15).

Binary logistic regression analyses disclosed a significant association between male gender (OR: 2.75, 95% CI: 1.34–5.65), LAI antipsychotic prescription (OR: 2.62, 95% CI: 1.84–3.74), aggressive behavior in REMS (OR: 1.63, 95% CI: 1.13–2.36), and antipsychotic polypharmacy (Table 2). High-dose antipsychotics were also associated with male gender (OR: 2.01, 95% CI: 1.02–3.95), LAI antipsychotic prescription (OR: 2.78, 95% CI: 1.95–3.97), and aggressive behavior in REMS (OR: 1.63, 95% CI: 1.12–2.36) (Table 3). A very high-dose antipsychotic prescription was associated with the use of LAI antipsychotic prescription (OR: 9.73, 95% CI: 4.88–19.43) but not with gender, diagnosis of schizophrenia spectrum disorder or personality disorder, and length of stay in REMS nor with aggressive behavior in REMS (Table 4).

**TABLE 2:** Binary logistic regression analysis of factors associated with antipsychotic polypharmacy.

Independent predictors	O.R.	C.I. 95%	*p*
Male gender	2.75	1.34–5.65	<0.01
LAI antipsychotic prescription	2.62	1.84–3.74	<0.001
Schizophrenia spectrum disorder and other psychoses diagnosis	0.93	0.59–1.49	ns
Length of stay in REMS	1.02	1.00–1.04	ns
Aggressive behavior in REMS, previous month (MOAS > 0)	1.63	1.13–2.36	<0.01
Personality disorder diagnosis	0.60	0.36–1.01	ns

*Cox & Snell R^2^ = 0.14; ns, not significant.*

**TABLE 3:** Binary logistic regression analysis of factors associated with high-dose antipsychotics prescription [prescribed daily dose (PDD)/defined daily dose (DDD) > 1.5].

Independent predictors	O.R.	C.I. 95%	*p*
Male gender	2.01	1.02–3.95	<0.05
LAI antipsychotic prescription	2.78	1.95–3.97	<0.001
Schizophrenia spectrum disorder and other psychoses diagnosis	1.45	0.92–2.30	ns
Length of stay in REMS	1.02	0.99–1.04	ns
Aggressive behavior in REMS, previous month (MOAS > 0)	1.63	1.12–2.36	0.01
Personality disorder diagnosis	0.83	0.50–1.38	ns

*Cox & Snell R^2^ = 0.10; ns, not significant.*

**TABLE 4:** Binary logistic regression analysis of factors associated with very high-dose antipsychotic prescription (PDD/DDD > 3).

Independent predictors	O.R.	C.I. 95%	*p*
Male gender	2.29	0.76–6.88	n.s.
LAI antipsychotic prescription	9.73	4.88–19.43	<0.001
Schizophrenia spectrum disorder and other psychoses diagnosis	1.32	0.67–2.60	n.s.
Length of stay in REMS	1.02	0.99–1.05	n.s.
Aggressive behavior in REMS, previous month (MOAS > 0)	1.52	0.92–2.53	n.s.
Personality disorder diagnosis	0.73	0.34–1.55	n.s.

*Cox & Snell R^2^ = 0.13; ns, not significant.*

First- (50.8%) or second-generation (49.2%) LAI antipsychotics were prescribed to *n* = 301 patients (48.6% of the sample excluding *n* = 62 missing data), including haloperidol decanoate (18.4%), paliperidone palmitate (15.0%), aripiprazole (4.8%), fluphenazine decanoate (3.4%), risperidone (3.1%), zuclopenthixol decanoate (2.9%), and olanzapine pamoate (0.9%). Since LAI antipsychotic prescription was associated with high and very high doses, as well as antipsychotic polypharmacy, we investigated the percentage of patients under high, very high antipsychotic regime, and antipsychotic polypharmacy for each LAI antipsychotics (Table 5). Those patients who received a first-generation LAI antipsychotic were more frequently under high-dose antipsychotics and polypharmacy than those who received a second-generation LAI antipsychotic (Table 6).

**TABLE 5:** Long-acting injectable (LAI) antipsychotics, high dose/very high-dose antipsychotic regimens, and antipsychotic polypharmacy.

Long-acting injectable (LAI) antipsychotic	High AP dose PDD/DDD > 1.5 n (%)	Very high AP dose PDD/DDD > 3 n (%)	Antipsychotic polypharmacy n (%)
No LAI antipsychotic (*n* = *318*)	120 (37.7%)	13 (4.1%)	108 (34.0%)
Haloperidol decanoate (*n* = *114*)	89 (78.1%)	34 (29.8%)	78 (68.4%)
Aripiprazole (*n* = *30*)	6 (20.0%)	1 (3.3%)	7 (23.3%)
Fluphenazine decanoate (*n* = *21*)	17 (81.0%)	11 (52.4%)	19 (90.5%)
Olanzapine pamoate (*n* = *6*)	4 (66.7%)	2 (33.3%)	3 (50.0%)
Paliperidone palmitate (*n* = *93*)	66 (71.0%)	30 (32.3%)	49 (52.7%)
Risperidone (*n* = *19*)	7 (36.8%)	2 (10.5%)	9 (47.4%)
Zuclopenthixol decanoate (*n* = *18*)	7 (38.9%)	1 (5.6%)	8 (44.4%)

*Classification refers to a comprehensive medication profile. p < 0.001 by chi-square for all the columns. N = 62 data were missing for LAI antipsychotic prescription.*

**TABLE 6:** Use of first or second-generation LAI antipsychotics in patients receiving high/very high-dose antipsychotics and antipsychotic polypharmacy.

Long-acting injectable (LAI) antipsychotic	High AP dose PDD/DDD > 1.5 n (%)	Very high AP dose PDD/DDD > 3 n (%)	Antipsychotic polypharmacy n (%)
1st generation LAI antipsychotics (*n* = 153)	113 (73.9%)	46 (30.1%)	105 (68.6%)
2nd generation LAI antipsychotics (*n* = 148)	83 (56.1%)	35 (23.6%)	68 (45.9%)
*p*	<0.01	ns	<0.001

*p-values by chi-square. ns, not significant.*

## Discussion

This retrospective study on the population of forensic psychiatric inpatients is the first describing antipsychotic prescription regime in the Italian population of REMS patients and among the few describing a large forensic psychiatric sample. Due to the clinical and criminological characteristics of the patients admitted to the REMS, the present sample of patients is characterized by high needs for care and rehabilitation as well as by high social dangerousness. Given the exclusively sanitary nature of REMS, we can hypothesize that antipsychotic therapy constitutes one of the main therapeutic means available to contain the symptomatological and behavioral aspects connected to social dangerousness. The current Italian system envisages REMS for patients with greater social dangerousness, possibly of limited duration, within a recovery path that aims to move to non-forensic facilities.

In our mixed-diagnosis sample, we found that 45.2% of the patients were prescribed antipsychotic polypharmacy. This result is similar to those emerged in mixed diagnosis studies conducted on acute psychiatric inpatients in the United Kingdom (50.5%) (Lelliott et al., 2002), and Portugal (41.6%) (Campos Mendes et al., 2016). Forty-three percent of antipsychotic polypharmacy also emerged in an Australian study of acute inpatients suffering from schizophrenia or schizoaffective disorder (John et al., 2014). A longitudinal Korean study found 37.1% (2005) and 48.3% (2010) of polypharmacy with antipsychotics in acute inpatients with schizophrenia. Lower percentages of antipsychotic polypharmacy (37.5%) were found at discharge from an acute psychiatric ward (McMillan et al., 2017) or in the community even in those under a community treatment order (32.0%) (Gisev et al., 2014b). However, the REMS patient population is not considered acute nor are REMS hospitals but community psychiatric facilities. The greater use of polypharmacy must obviously respond to different clinical needs.

The multivariate analysis we conducted disclosed an association between antipsychotic polypharmacy and male gender (OR: 2.75 and 95% CI: 1.34–5.65), LAI antipsychotic prescription (OR: 2.62 and 95% CI: 1.84–3.74), and aggressive behavior in REMS (OR: 1.63 and 95% CI: 1.13–2.36). Some studies on different populations have not shown an association between male gender and antipsychotic polypharmacy (Gisev et al., 2014b; Campos Mendes et al., 2016; McMillan et al., 2017), while others found such results. Others found an association with the duration of hospitalization (Lelliott et al., 2002; Connolly and Taylor, 2014). The result of an association between polypharmacy with antipsychotics and aggressive behavior during the stay in a forensic facility does not appear to have been previously described. The association between this behavioral variable and polypharmacy with antipsychotics raises the hypothesis that psychiatrists might have weighted the behavioral and security issues in their therapeutic choices. Despite the diagnosis of schizophrenia spectrum disorder predicted antipsychotic polypharmacy in previous studies (Lelliott et al., 2002), we found no association in the REMS population nor with the diagnosis of personality disorder.

More than half (50.8%) of the patients in our study received high-dose antipsychotics, and a higher rate than those found in the study by Roh et al. (30.4% in 2005 and 14.8% in 2010) (Roh et al., 2014) conducted on a sample of acute inpatients, using the same definition of high-dose antipsychotics (PDD/DDD > 1.5). Of note, 14% of the patients were prescribed very high-dose antipsychotics, corresponding to a dosage three times higher than the DDD.

A significant association between the prescription of an LAI antipsychotic and high dose (OR: 2.78 and 95% CI: 1.02–3.95) and very high-dose antipsychotics (OR: 9.73, 95% CI: 4.88–19.43) prescription emerged in the respective multivariate analyses. Interestingly, the aggressive behavior of patients in the REMS, which is manifested during the last month before data collection, was also associated with high-dose antipsychotics, a result that seems to confirm the abovementioned hypothesis for antipsychotic polypharmacy. Diagnosis of psychosis or personality disorder proved no association with high dose nor with very high-dose antipsychotics. Altogether, our results indicate that the qualitative-quantitative therapeutic choices of antipsychotics made on the REMS patient population are closer to those of acute psychiatric patients rather than in community rehabilitation.

The use of an LAI antipsychotic was associated with polypharmacy, high dose, and very high-dose antipsychotics. Interestingly, we found that first-generation LAI antipsychotics were more frequently associated with high-dose antipsychotics and polypharmacy than those who received a second-generation LAI antipsychotic (Table 6). The use of first-generation drugs, in the context of high dosages of antipsychotics and polypharmacotherapy, deserves reflection on the reasons for therapeutic choices that also seem to be linked to the need for behavioral control and that hardly fall within the prescriptive guidelines. The Italian law provides that in the REMS, safety provision is completely entrusted to healthcare workers. There are moreover no treatment facilities providing a higher level of safety, for those aggressive patients who may be treatment-resistant or present poor treatment compliance. In a forensic psychiatric system such as that of REMS, the use of high-dose antipsychotics and polypharmacy could be among the few options available to the psychiatrist who is required by the law to treat patients and reduce their social dangerousness. This hypothesis is also linked to the evidence that the prescribing psychiatrists also bear the responsibility for the safety of other patients, as well as health personnel (Catanesi, 2017). A question that is still unanswered is whether this patient population is adequately informed and whether patients have the mental capacity to consent to such treatment (Mandarelli et al., 2014, 2018; Carabellese et al., 2018).

The present observational retrospective study has limitations. The data collection period is close to that of the opening of the REMS in Italy; thus, it is possible that it occurred during a phase of adaptation of a new and complex system, with possible repercussions on the data we collected. The choice to use the DDD method was made because they are internationally accepted measures, available for almost all antipsychotic drugs, including older antipsychotics, as we found a large variety of molecules in the study sample. This method has limitations; however, other methods (Leucht et al., 2016) may produce slightly different results for high-dose antipsychotics. Among the strengths of the study, it is worth noting the size of the sample of patients, greater than previous similar studies, to be considered representative of the new Italian REMS forensic psychiatric population.

## Conclusion

This study highlights a frequent use of antipsychotic polypharmacy and high-dose antipsychotics in the REMS population, comparable with or greater than acute psychiatric inpatients. Aggressive behavior in the REMS proved to be associated with such therapeutic choices, a result that deserves attention and that could be justified by the attempt to obtain a pharmacological control and prevention of aggressive and violent behavior. These therapeutic choices are, however, burdened by an increased risk of side effects and possible health risks and are often unaligned with the prescribing clinical guidelines. The use of polypharmacy and high-dose antipsychotics may also depend on regulatory and organizational aspects of the REMS system, including variability in structures, lack of a common model of care, and lack of stratified therapeutic security (Kennedy, 2021).

## Data Availability Statement

The raw data supporting the conclusions of this article will be made available by the authors, without undue reservation.

## Ethics Statement

The studies involving human participants were reviewed and approved by University of Bari Aldo Moro. Written informed consent for participation was not required for this study in accordance with the national legislation and the institutional requirements.

## Author Contributions

GM and RC conceived of the study, analyzed data, and wrote the original draft. FC and GS interpreted data and critically revised the manuscript. RC gave the final approval of the version to be published. All authors contributed to the article and approved the submitted version.

## Conflict of Interest

The authors declare that the research was conducted in the absence of any commercial or financial relationships that could be construed as a potential conflict of interest.

## Publisher’s Note

All claims expressed in this article are solely those of the authors and do not necessarily represent those of their affiliated organizations, or those of the publisher, the editors and the reviewers. Any product that may be evaluated in this article, or claim that may be made by its manufacturer, is not guaranteed or endorsed by the publisher.
